# Gut‐liver axis: Potential mechanisms of action of food‐derived extracellular vesicles

**DOI:** 10.1002/jev2.12466

**Published:** 2024-06-17

**Authors:** Sitong Zhang, Qiyue Wang, Daniel En Liang Tan, Vritika Sikka, Cheng Han Ng, Yan Xian, Dan Li, Mark Muthiah, Nicholas W. S. Chew, Gert Storm, Lingjun Tong, Jiong‐Wei Wang

**Affiliations:** ^1^ Department of Surgery, Yong Loo Lin School of Medicine National University of Singapore Singapore Singapore; ^2^ Nanomedicine Translational Research Programme, Centre for NanoMedicine, Yong Loo Lin School of Medicine National University of Singapore Singapore Singapore; ^3^ Jinan Central Hospital Shandong First Medical University & Shandong Academy of Medical Sciences Jinan China; ^4^ Medical Science and Technology Innovation Center Shandong First Medical University & Shandong Academy of Medical Sciences Jinan China; ^5^ Division of Gastroenterology and Hepatology, Department of Medicine National University Hospital Singapore Singapore; ^6^ Department of Food Science and Technology, Faculty of Science National University of Singapore Singapore Singapore; ^7^ National University Centre for Organ Transplantation National University Health System Singapore Singapore; ^8^ Department of Cardiology National University Heart Centre National University Health System Singapore Singapore; ^9^ Cardiovascular Research Institute (CVRI) National University Heart Centre Singapore (NUHCS) Singapore Singapore; ^10^ Department of Physiology, Yong Loo Lin School of Medicine National University of Singapore Singapore Singapore

**Keywords:** food‐derived extracellular vesicles, gut barrier, gut microbiota, gut‐liver axis, metabolic disease, milk‐derived extracellular vesicles, nanomedicine, oral drug delivery system, plant‐derived extracellular vesicles, probiotics

## Abstract

Food‐derived extracellular vesicles (FEVs) are nanoscale membrane vesicles obtained from dietary materials such as breast milk, plants and probiotics. Distinct from other EVs, FEVs can survive the harsh degrading conditions in the gastrointestinal tract and reach the intestines. This unique feature allows FEVs to be promising prebiotics in health and oral nanomedicine for gut disorders, such as inflammatory bowel disease. Interestingly, therapeutic effects of FEVs have recently also been observed in non‐gastrointestinal diseases. However, the mechanisms remain unclear or even mysterious. It is speculated that orally administered FEVs could enter the bloodstream, reach remote organs, and thus exert therapeutic effects therein. However, emerging evidence suggests that the amount of FEVs reaching organs beyond the gastrointestinal tract is marginal and may be insufficient to account for the significant therapeutic effects achieved regarding diseases involving remote organs such as the liver. Thus, we herein propose that FEVs primarily act locally in the intestine by modulating intestinal microenvironments such as barrier integrity and microbiota, thereby eliciting therapeutic impact remotely on the liver in non‐gastrointestinal diseases via the gut‐liver axis. Likewise, drugs delivered to the gastrointestinal system through FEVs may act via the gut‐liver axis. As the liver is the main metabolic hub, the intestinal microenvironment may be implicated in other metabolic diseases. In fact, many patients with non‐alcoholic fatty liver disease, obesity, diabetes and cardiovascular disease suffer from a leaky gut and dysbiosis. In this review, we provide an overview of the recent progress in FEVs and discuss their biomedical applications as therapeutic agents and drug delivery systems, highlighting the pivotal role of the gut‐liver axis in the mechanisms of action of FEVs for the treatment of gut disorders and metabolic diseases.

## Graphical abstract

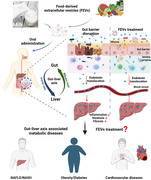

## 1 INTRODUCTION

Gut‐liver axis enables interaction between the gut and liver through the portal vein and bile duct. Via the portal vein, the liver receives 75% of its blood supply from the intestines (Henao‐Mejia et al., 2013; Tripathi et al., 2018). Accompanied by the integration of signals generated by both internal and external factors, including diet, genes, and environment, this communication process is regulated by two main components: gut microbiota and gut barrier (Albillos et al., 2020). The gut microbiota can impact the liver directly through bacterial metabolites or indirectly through modulating the intestinal barrier. An alteration of specific types of gut bacteria may lead to an imbalance of metabolites, thereby affecting intestinal health status or disease progression (Tripathi et al., 2018). Moreover, the gut microbiota may contribute to liver diseases through various mechanisms, including intestinal hyperpermeability, chronic systemic inflammation and alterations in bacterial metabolism (Schwenger et al., 2019). Apart from gut microbiota, the gut barrier also constitutes an essential integrity component of the gut. Composed of intestinal mucosal and vascular barrier, the gut barrier is the functional and anatomical structure that mediates interactions between the gut and the liver. It limits the systemic dissemination of microbes and toxins while allowing nutrients to access the circulation and reach the liver (Albillos et al., 2020). Multiple factors can lead to disruption of the gut barrier, such as unhealthy diet, dysbiosis, and chronic inflammation, which can further cause leakage of viable bacteria, bacterial components, and bacterial metabolites, thereby leading to liver injury. In fact, gut disorders have been associated with multiple metabolic diseases, including non‐alcoholic fatty liver disease (NAFLD), obesity, diabetes and cardiovascular disorders (Martel et al., 2022; Ohtani & Kawada, 2019). The gut microbiome‐associated pathogenesis provides an opportunity to explore alternative therapeutic strategies targeting the whole flora or some specific bacterial species for the treatment of metabolic and gastrointestinal (GI) diseases. For instance, by modulating the gut microbiota, fecal microbiota transplantation (FMT), Rifaximin and Vancomycin are therapeutically efficacious in alleviating inflammatory bowel disease (IBD), hepatic encephalopathy and primary sclerosing cholangitis, respectively (Damman et al., 2018; Haussinger et al., 2022; Imdad et al., 2023). Given the close interconnection between the gut microbiome, intestine, and liver, modulating the gut barrier and gut microbiome by controlling diet, lifestyle or pharmaceutical interventions is potentially a new strategy to treat metabolic disorders.

Extracellular vesicles (EVs) are released by almost all living cells and are involved in various (patho)physiological processes (Huang et al., 2021). Food‐derived EVs (FEVs) have gained more attention in recent years due to emerging evidence correlating diet to gut health, and the unique features of FEVs such as their resistance to the degrading gastric environment (Hasan & Yang, 2019; Nova et al., 2022). FEVs are derived from a variety of food sources, such as milk, plants, and probiotics. FEVs are natural nanoparticles ranging from 30 to 500 nm, carrying bioactive molecules (e.g., proteins and miRNAs) for cell‐to‐cell communication (Munir et al., 2020). FEVs are often administered orally, whereas other administration routes are relatively rare and do not leverage on the gut‐liver axis. Following oral administration, FEVs can be taken up by colon immune cells and epithelial cells in the colon (Amagase et al., 2001; Keller et al., 2006; Milner, 2004; Tong et al., 2023). Currently, most original studies and literature reviews focus on the modulatory effects of FEVs on gut disorders with limited attention to liver disorders and beyond. Interestingly, recent studies suggest that oral administration of FEVs protects the liver from injury and improves metabolic conditions (Du et al., 2021; Tong et al., 2023). These findings give hope to FEVs‐based therapeutic strategies for liver diseases and metabolic disorders. Despite the exciting and potentially impactful observations, the underlying mechanisms of action remain elusive. Hence, in this review, we aim to provide an overview of recent progress and biomedical applications of FEVs in metabolic diseases and discuss their potential mechanisms of action with a focus on the gut‐liver axis.

## 2 GUT‐LIVER AXIS

**FIGURE 1:**
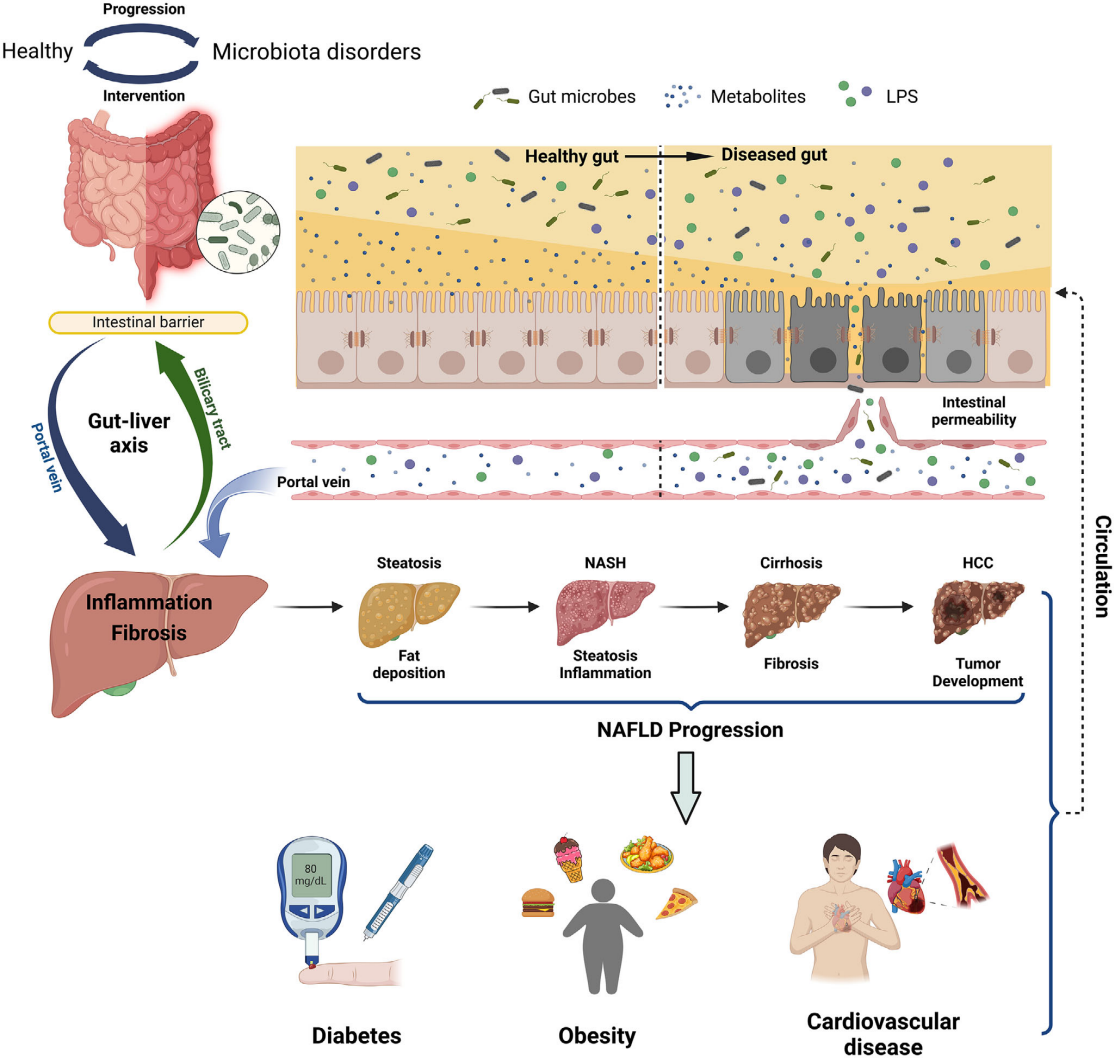
The gut‐liver axis in healthy conditions and NAFLD‐associated metabolic disorders. Gut microbiota alterations can affect the production of a variety of metabolites, such as short‐chain fatty acids (SCFAs), bile acids (BAs), and aromatic amino acids (AAAs). Under certain disease conditions, the gut barrier integrity is disrupted, leading to an increase in intestinal permeability. The leakage of detrimental compounds to blood circulation may induce inflammation in the liver, thereby deteriorating the progression of liver diseases and associated metabolic diseases, such as diabetes, obesity and cardiovascular disease. NASH, non‐alcoholic steatohepatitis; HCC, hepatocellular carcinoma; NAFLD, non‐alcoholic fatty liver disease.

The gut‐liver axis is a reciprocal interaction between the gut and liver established by the blood circulation, portal vein, and the bile duct. As illustrated in Figure 1, in this axis, gut‐derived products are directly transported via the portal vein to the liver with the liver secreting bile acids to the intestine. Under healthy status, intestinal components will pass through multiple layers of the gut barrier to reach the liver through the gut‐liver axis, ensuring microbial transfer in a controlled manner (Albillos et al., 2020). On the other hand, the gut barrier is crucial in maintaining gut metabolic and immune homeostasis. The epithelial barrier is mainly maintained by tight junctions, which are regulated by tight junction proteins such as claudins, junctional adhesion molecules and zonula occludens. Intestinal immune cells such as Th17 cells and T cells, mucus, and soluble mediators such as IgA, also contribute to the integrity of the gut barrier and anti‐infectious responses (Albillos et al., 2020; Woof & Kerr, 2006).

### 2.1 Gut microbiota in the gut‐liver axis

Gut microbiota is one of the key components of the gut‐liver axis (Figure 1). During physiological activities, the intestine and liver secrete bioactive substances that either are transported between the two organs or act on the intestinal flora. In addition, intestinal microbes can produce various metabolites that are absorbed by the intestinal tissue and/or influence the intestinal barrier permeability. The absorbed metabolites can be transported through the portal vein to the liver, and thereby influencing hepatic function (Starkel & Schnabl, 2016). Furthermore, systemic circulation extends the gut‐liver axis via transporting liver metabolites, such as free fatty acids (FFAs) and ethanol metabolites, to the intestine. All these metabolite exchanges may pose an impact on the intestinal barrier and affect the gut‐liver axis functionality (Tripathi et al., 2018).

Apart from producing metabolites to mediate communication between the gut and liver, gut microbes per se and their metabolites can shuffle from the gut to the liver (Anand & Mande, 2022). In fact, gut eubiosis is maintained by limiting bacterial overgrowth, a process mediated by bile salts and endogenous antimicrobial molecules which are transported from the liver to the intestine through the biliary tract and blood circulation (Starkel & Schnabl, 2016). Gut microbiota affects the plasma levels of a variety of metabolites, including short‐chain fatty acids (SCFAs), bile acids (BAs) and aromatic amino acids (AAA) derivatives, which mediate the interaction between the gut and liver. Acetic, propionic and butyric acids are the major SCFA products of carbohydrate fermentation by gut bacteria (Leung et al., 2016; Nyangale et al., 2012). AAA, including tryptophan (Trp), phenylalanine (Phe), and tyrosine (Tyr), produced by proteolysis in the GI tract are also catabolized by the gut microbiome. This yields numerous metabolites related to immune, metabolic, and neuronal responses at local and distant sites (Liu et al., 2020b).

### 2.2 Gut barrier in the gut‐liver axis

The intestinal barrier is composed of three structured layers. The first layer is the mucus which contains mucin secreted by the intestinal goblet cells. The mucus layer hosts the microbes and prevents direct contact between the bacteria and the epithelial cells (Johansson et al., 2013). The epithelial barrier is composed of specialized epithelial cells linked by intercellular junctions to seal the GI space and prevent the paracellular passage of bacteria (Martin‐Mateos & Albillos, 2021). Immune cells in the lamina propria (LP) at the other side of the epithelial barrier constitute the immune barrier of the gut, which can generate direct defensive immune responses against the pathogens crossing the intestinal epithelium. The three layers work in synergy, making the gut barrier a key regulator in the gut‐liver axis to control transportation from the gut to the liver and systemic circulation. Under healthy conditions, the epithelial tight junction is intact and the gut barrier can absorb water and nutrients while hindering penetration of harmful intestinal contents to the blood circulation and internal organs (Spadoni et al., 2017, 2015). However, when gut microbiota dysregulation occurs, the gut barrier may be disrupted (Cao et al., 2021), leading to a leaky gut characterized by increased intestinal permeability (Figure 1). The leaky gut can be worsened by damage to the gut‐vascular barrier (Tilg et al., 2016). In summary, translocation of inflammatory microbial products to the liver through the leaky gut barrier may induce hepatic inflammation and deteriorate liver disorders via the gut‐liver axis.

## 3 GUT‐LIVER AXIS IN DISEASE

### 3.1 Gut‐liver axis in gastrointestinal disorders

**FIGURE 2:**
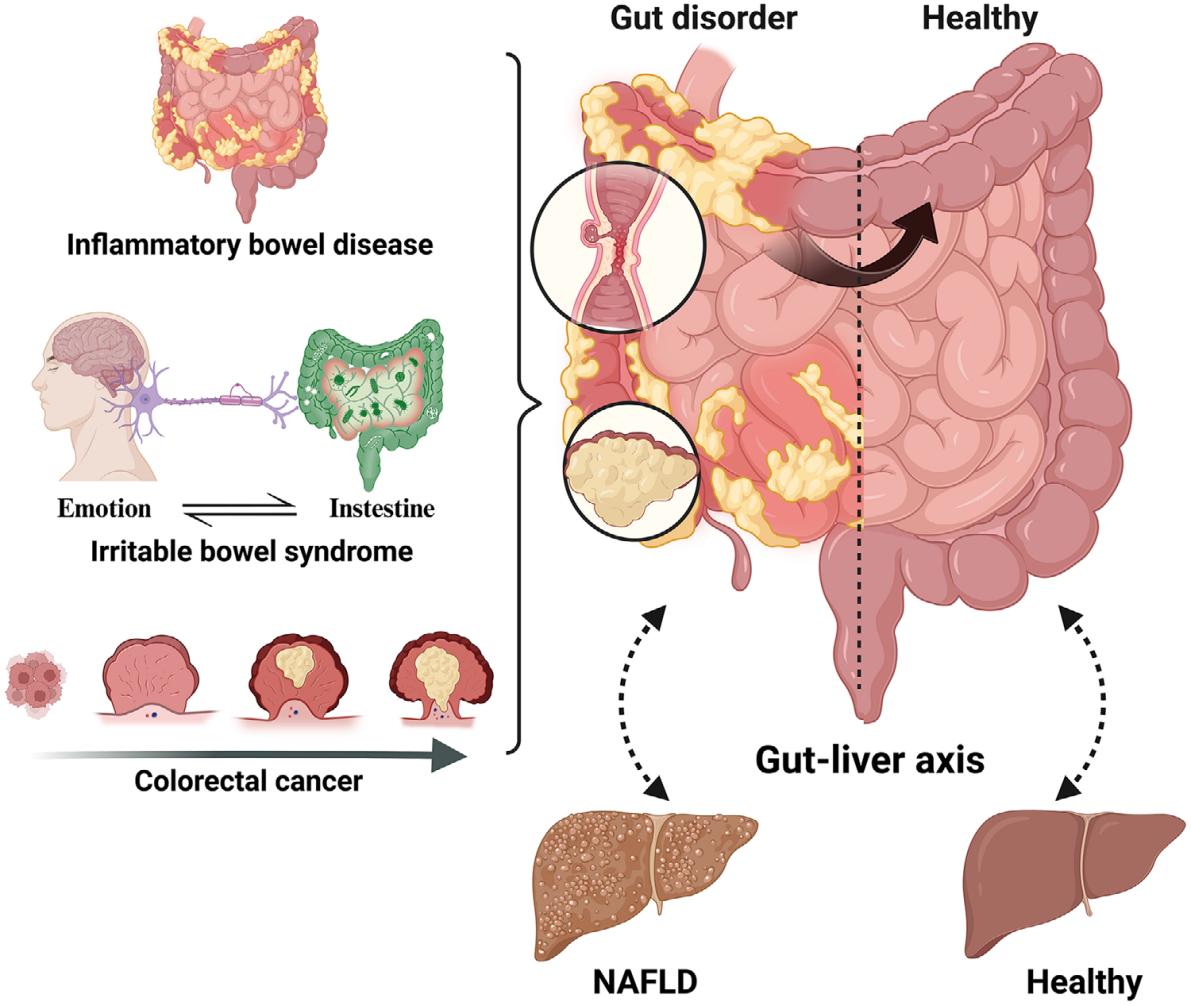
Gut‐liver axis in intestinal disorders. Gut microbiota and gut barrier are disturbed in intestinal diseases, such as inflammatory bowel disease, irritable bowel syndrome, and colorectal cancer, which may induce or deteriorate liver injury and diseases such as NAFLD. In turn, liver disorders may modulate gut conditions through the gut‐liver axis. NAFLD, non‐alcoholic fatty liver disease.

Gut microbiota co‐evolves with the host to form a mutually beneficial relationship (Backhed et al., 2005). An imbalance of gut microbiota with decreased microbial diversity and increased proinflammatory species is defined as dysbiosis (Martinez et al., 2021). In response to certain dietary challenges or mental stress, gut bacterial species residing within the intestinal mucus layer may be altered (Carding et al., 2015), thereby influencing host cellular homeostasis and triggering inflammatory response either through direct contact with host cells or indirect communications via bacterial metabolites. This communication between gut microbiome and the host may also be mediated by EVs derived from food, bacteria, or host cells despite the evidence being rather preliminary (Tong et al., 2021a). More importantly, dysbiosis has been associated with intestinal disorders, such as IBD, irritable bowel syndrome (IBS) and colorectal cancer (Figure 2) (Carding et al., 2015).

Increasing evidence suggests the interaction between GI disorders and liver diseases, bringing more attention to the centrepiece of this process, the gut‐liver axis. Dysbiosis may induce gut inflammation and gut barrier disruption, and subsequently pose stress on the liver or even cause liver disorders. In turn, liver disorders can affect gut diseases by modulating bile acid circulation (Figure 2). For example, liver diseases, such as nonalcoholic steatohepatitis (NASH), can disturb the enterohepatic circulation of BAs, thus inducing dysbiosis and a leaky gut (Hylemon et al., 2021; Smirnova et al., 2022). In both human patients and animal models, IBD can cause or aggravate hepatic inflammation, and vice versa, through the gut‐liver axis (Bessissow et al., 2016; Likhitsup et al., 2019; Shen et al., 2021; Tong et al., 2023). Apart from IBD, the co‐incidence of non‐alcoholic fatty liver disease (NAFLD) in patients with IBS has drawn much attention due to their shared risk factors and pathophysiology. Interestingly, emerging evidence suggests that patients with IBS are more likely to develop NAFLD (Grant et al., 2022; Oka et al., 2020). Moreover, the liver is the most common site of tumour metastasis for patients with colorectal cancer, and in turn a fatty liver promotes colorectal cancer liver metastasis (Martin et al., 2020; Wang et al., 2023b). The close association between these GI disorders and liver diseases further supports the critical role of gut‐liver axis.

### 3.2 Gut‐liver axis in liver diseases

The liver is the initial site for detoxification of gut microbial products collected from the portal vein. The gut‐liver axis has been implicated in multiple liver diseases, including alcohol‐associated liver disease (ALD), NAFLD, NASH, cirrhosis and hepatocellular carcinoma (HCC) (Bajaj et al., 2015; Dubinkina et al., 2017; Muthiah et al., 2022; Pezzino et al., 2022). As known, the majority of non‐viral liver diseases are related to metabolic disorders. Unhealthy lifestyles, including long‐term alcohol exposure, excessive caloric intake, as well as limited physical activity and exercise, can pose stress on gut microbiota, thus triggering dysbiosis (Albillos et al., 2020). Dysbiosis is often accompanied by intestinal barrier disruption (or a leaky gut), leakage of endotoxin, alterations in bile acid profiles, and reduction of choline levels (Song & Zhang, 2022). These factors facilitate the permeation of the gut microbiome, its metabolites, and bacterial EVs across the intestinal barrier to enter the portal system, thereby aggravating liver disease progression (Ding et al., 2019; Fan & Pedersen, 2021). For instance, continuous exposure of the liver to microbial metabolites triggers hepatic immune responses and deteriorates hepatic steatosis (Hoyles et al., 2018; Krishnan et al., 2018; Manna et al., 2011; Natividad et al., 2018).

In addition to the direct toxic effects of ethanol on liver parenchymal cells, disturbed gut microbiota and disrupted intestinal barrier also contribute to the pathogenesis of ALD (Yan et al., 2011). Notably, dysbiosis starts before liver fibrosis in ALD (Bull‐Otterson et al., 2013; Chen et al., 2015; Seki et al., 2007). It was reported that gut bacterial endotoxin levels increased in the blood circulation after excessive alcohol intake followed by activation of liver immune cells (Petrasek et al., 2010; Uesugi et al., 2001). Distinct from ALD, NAFLD is often caused by a lack of exercise and unhealthy eating habits. More importantly, gut microbiota transplantation, probiotics, prebiotics and symbiotic supplementation ameliorate liver disorders in both patients and animal models (Asgharian et al., 2016; Lata et al., 2007; Xie & Halegoua‐DeMarzio, 2019), indicating critical roles of gut microbiota in NAFLD/NASH. While dysbiosis and intestinal barrier hyperpermeability are involved in and lead to hepatic inflammation in both ALD and NAFLD (Safari & Gerard, 2019), ALD and NAFLD have subtle differences in terms of intestinal microbial composition, gut permeability, BAs, ethanol and choline metabolites (Hoyles et al., 2018; Loomba et al., 2017; Luck et al., 2015). In particular, altered microbial metabolism of BAs is closely associated with hepatic steatosis and insulin resistance (Leung et al., 2016). In cirrhosis, the leaky gut constitutes an important pathogenetic factor for major complications in the patients. Interestingly, pathological bacterial translocation tends to induce liver inflammation, leading to lowered synthesis of BAs (Fukui, 2021). Nonetheless, both dysbiosis and intestinal barrier dysfunction are directly associated with compensated cirrhosis and affect the frequency and degree of complications in decompensated cirrhosis (Bajaj et al., 2018; Du Plessis et al., 2013).

### 3.3 Gut‐liver axis in other metabolic diseases

The gut‐liver axis is also involved in other metabolic diseases, including obesity, type 2 diabetes (T2D) and cardiovascular diseases (CVD). Gut microbiota has been reported to regulate obesity through multiple pathways, such as energy absorption, fat storage, central appetite, chronic inflammation, and circadian rhythms (Liu et al., 2021b). For instance, butyrate, a SCFA generated by fermentation of dietary fibres, can increase fatty acid oxidation in adipocytes, thereby potentially prohibiting weight gain in obesity (Kim et al., 2019; Liu et al., 2021b). Interestingly, the abundance of butyrate‐producing bacteria is reduced and the opportunistic pathogens increased in the gut of T2D patients (Qin et al., 2012). These alterations in the gut microbiota may contribute to chronic inflammation, insulin resistance and metabolic dysfunction associated with the development of T2D. Moreover, a compromised gut barrier in T2D patients may lead to translocation of gut bacteria and their byproducts into the systemic circulation, resulting in inflammatory responses and insulin resistance (Yang et al., 2021). Furthermore, dysregulation of the gut‐liver axis influences hepatic lipid metabolism, leading to fat accumulation in the liver and contributing to insulin resistance (Manilla et al., 2023). Gut microbiota dysbiosis, trimethylamine N‐Oxide (TMAO) pathway, dysregulated bile acid metabolism, inflammation, intestinal barrier disruption, reduced production of SCFAs, liver dysfunction, and metabolic syndrome have all been observed in CVD (Rahman et al., 2022). Given that alterations in gut microbiome and gut barrier disruption occur in a variety of liver diseases including NAFLD, it is not surprising that the presence of NAFLD increases the incidence of CVD through the production of TMAO (Anand & Mande, 2022). Overall, the gut‐liver axis plays a critical role in metabolic regulation and dysregulation of the gut‐liver axis has been linked to various types of metabolic disorders. Understanding the interactions between the gut and the liver helps to uncover novel pathogenic mechanisms underlying various seemingly unrelated metabolic disorders. As a result, modulating the gut microbiota and improving gut barrier function in the gut‐liver axis constitute a new strategy to tackle metabolic diseases.

## 4 FOOD‐DERIVED EXTRACELLULAR VESICLES

EVs derived from food, including plants, probiotics and milk of humans and various animals, reach the intestine following oral administration and have been reported to pose biological effects on the gut microbiota and gut barrier. Emerging evidence indicates that FEVs have therapeutic potential in intestinal diseases and metabolic disorders via effects on the gut‐liver axis.

### 4.1 Sources and characterization

**FIGURE 3:**
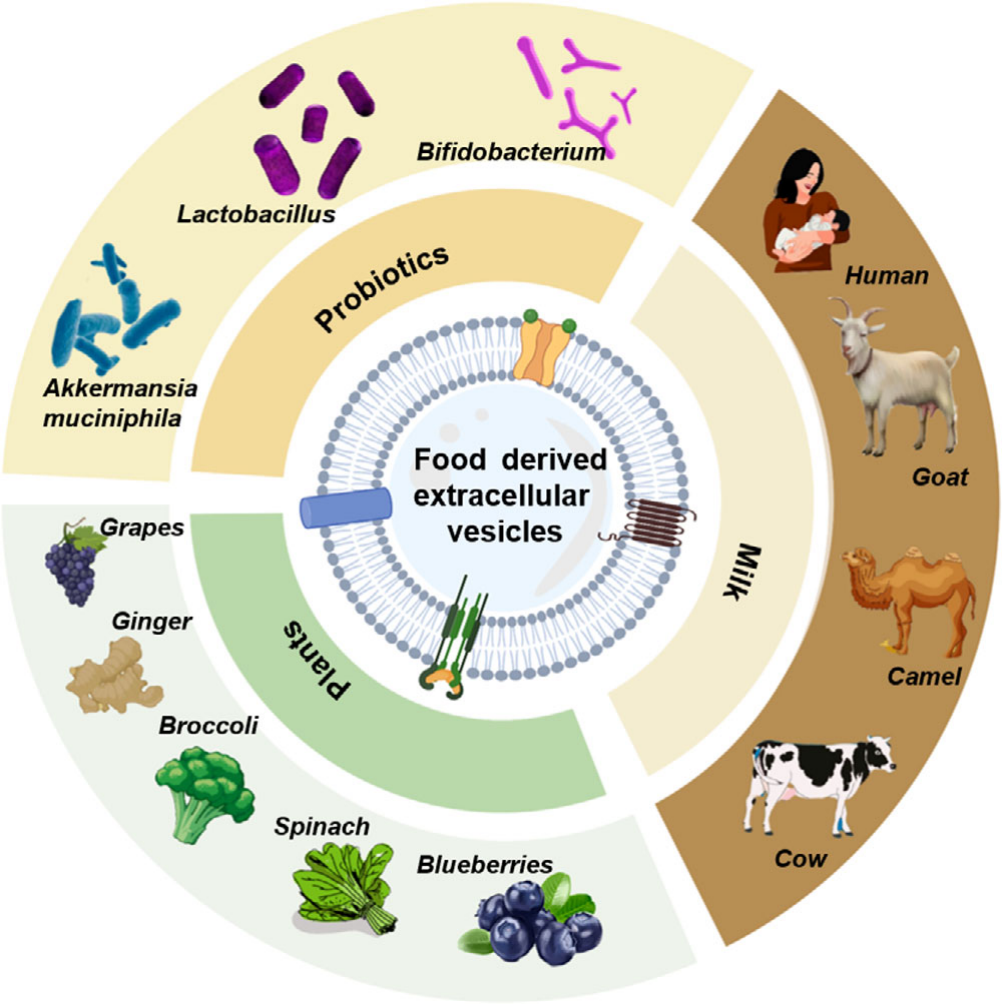
Known sources of food‐derived extracellular vesicles.

FEVs are a class of nanoscale membranous vesicles derived from food and carry biomolecules for cell‐to‐cell communication. FEVs can be obtained from a variety of sources, including milk (e.g., from humans, cows, goats, and pigs), vegetables (e.g., ginger and garlic), fruits (e.g., grapefruit and lemon) and probiotics (e.g., *Bifidobacterium longum*, *Clostridium butyricum*, and *Lactobacillus plantarum*) (Tables 1 and 2, Figure 3). It should be noted that EVs derived from other bacteria other than probiotics, such as outer membrane vesicles (OMVs), are not considered as FEVs. In comparison with EVs of other origins, FEVs exhibit superiority in terms of source accessibility, biosafety, GI stability and suitability for oral administration.

**TABLE 1:** Recent advances in FEVs‐based therapy for gut disorders.

EV source	Function	Disease	Subject	Administration route	Active ingredients	Outcomes	Ref.
Bovine milk	Therapeutic	NA	Mice	Oral gavage	Not reported	mEVs alter bacterial communities in murine cecum. The abundance of three phyla, seven families and 52 operational taxonomic units is different in the ceca between mice fed on mEVs/RNA‐depleted and mEVs/RNA‐sufficient diets.	Zhou et al. (2019)
Bovine milk	Therapeutic	NA	Mice	Oral gavage	Not reported	mEVs modulate intestinal immunity and the composition of gut microbiome and SCFAs.	Tong et al. (2020)
Bovine milk	Therapeutic	Ulcerative colitis (UC)	Mice	Oral gavage	Not reported	Oral administration of mEVs alleviates UC by regulating intestinal immune homeostasis via inhibiting TLR4‐NF‐κB and NLRP3 signaling pathways, restoring Treg/Th17 cell balance, and reshaping the gut microbiota.	Tong et al. (2021a)
Bovine milk	Therapeutic	UC	Mice	Oral gavage	Not reported	mEVs attenuate UC by optimizing gut microbiota abundance and by manipulating intestinal gene expression, implying their application potential for UC prevention.	Du et al. (2022)
Bovine milk	Therapeutic	Malnutrition	Mice	Oral gavage	Not reported	Low protein diet‐fed mice develop intestinal villus atrophy and barrier dysfunction. mEVs improve intestinal permeability, intestinal architecture and cellular proliferation in this malnutrition animal model.	Maghraby et al. (2021)
Bovine milk	Delivery vector	NA	Mice	i.v. injection and oral gavage	miRNAs	miRNAs delivered by mEVs accumulate in the intestinal mucosa, spleen, liver, heart and brain.	Manca et al. (2018)
Porcine milk	Therapeutic	NA	Mice	Oral gavage	Not reported	mEVs promote intestinal cell proliferation and intestinal tract development.	Chen et al. (2016)
Broccoli	Therapeutic	UC	Mice	Oral gavage	Sulforaphane (SFN)	Oral administration of broccoli EVs reduces mucosa inflammation and attenuates colonic shortening. SFN delivered by broccoli EVs alleviates mouse UC.	Deng et al. (2017)
Ginger	Therapeutic	UC and UC‐associated cancer	Mice	Oral gavage	Not reported	Ginger EVs alleviate acute UC, enhance intestinal repair, and prevent chronic UC and UC‐associated cancer.	Zhang et al. (2016a)
Ginger	Delivery vector	Colorectal cancer	Mice	i.v. injection	Doxorubicin (Dox)	Ginger EVs can serve as a delivery platform for the therapeutic agent Dox to treat colon cancer. Ginger EVs show a better pH‐dependent drug‐release profile than commercially available liposomal‐Dox.	Zhang et al. (2016b)
Ginger	Delivery vector	UC and UC‐associated cancer	Mice	Oral gavage	siRNA against CD98	Ginger EVs loaded with siRNA‐CD98 effectively decrease CD98 expression, thus hindering the progression of UC and UC‐associated cancer.	Zhang et al. (2017)
Ginger, carrot, grapefruit and grape	Therapeutic	Anti‐inflammatory response	Mice	Oral gavage	Not reported	Plant EVs inhibit inflammation by inducing nuclear translocation of macrophage Nrf2 and intestinal Wnt/TCF4 activation.	Mu et al. (2014)
Grape	Therapeutic	UC	Mice	Oral gavage	Not reported	Grape EVs can penetrate the intestinal mucus barrier and be taken up by intestinal stem cells. Oral administration of grape EVs alleviates UC via induction of intestinal stem cells.	Ju et al. (2013)
Grape	Delivery vector	NA	Rats	Oral gavage	Not reported	Grape EVs can survive gastric digestive conditions. Grape EVs are taken up by intestinal stem cells.	Rahimi et al. (2018)
Grapefruit	Therapeutic	UC	Mice	Oral gavage	Not reported	Grapefruit EVs alleviate DSS‐induced UC.	Wang et al. (2014)
Grapefruit	Delivery vector	UC	Mice	Oral gavage	Methotrexate (MTX)	Oral delivery of MTX by grapefruit EVs enhances its therapeutic efficacy in DSS‐induced UC while lowering its toxicity.	Wang et al. (2014)
*Lactobacillus*	Therapeutic	IBD	Mice	Oral gavage	Not reported	*Lactobacillus* EVs inhibit tumour necrosis factor‐α‐induced inflammation in Caco‐2 cells and alleviate IBD progression in vivo.	Seo et al. (2018)
*Lactobacillus rhamnosus GG*	Therapeutic	UC	Mice	Oral gavage	Not reported	*Lactobacillus* EVs alleviate colonic tissue damage, colon shortening and intestinal inflammation. *Lactobacillus* EVs restore the gut microbiota and associated metabolism pathways.	Tong et al. (2021b)
*Lactobacillus plantarum Q7*	Therapeutic	UC	Mice	Oral gavage	Not reported	*Lactobacillus* EVs restore diversity of gut microbiota and alleviate DSS‐induced UC.	Hao et al. (2021)
*Clostridium butyricum*	Therapeutic	UC	Mice	Oral gavage	Not reported	*Clostridium butyricum* EVs protect gut barrier function and improve gut microbiota homeostasis in UC.	Ma et al. (2022)

**TABLE 2:** Recent advances in FEVs‐based therapy for liver disorders.

EV source	Function	Disease	Subject	Administration route	Active ingredients	Outcomes	Ref.
Bovine milk	Therapeutic	NASH	Mice	Oral gavage	miRNA‐148a	Oral administration of mEVs restores gut barrier integrity and prevents endotoxin translocation into the liver in chemical‐induced experimental UC and diet‐induced nonalcoholic steatohepatitis (NASH), thus alleviating gut disorders and associated liver inflammation, and NASH.	Tong et al. (2023)
Bovine and human milk	Therapeutic	Liver fibrosis and cirrhosis	Mice	Oral gavage	Not reported	mEVs alleviate liver fibrosis by inhibiting proliferation and activation of hepatic stellate cells (HSC).	Reif et al. (2022)
Ginger	Therapeutic	Alcohol‐induced liver damage	Mice	Oral gavage	Shogaol	Oral administration of ginger EVs enhances liver detoxification and antioxidant capability, thereby alleviating alcohol‐induced liver damage.	Zhuang et al. (2015)
Tea Leaves	Therapeutic	Liver fibrosis	Mice	Oral gavage	Not reported	Tea EVs alleviate CCl_4_‐induced liver fibrosis by inhibiting HSC activation.	Gong et al. (2022)
Shiitake mushroom	Therapeutic	Fulminant hepatic failure (FHF)	Mice	i.p. injection	Not reported	Shiitake mushroom EVs protect mice from GalN/LPS‐induced acute liver injury by inhibiting NLRP3 inflammasome activation.	Liu et al. (2020a)
*Akkermansia muciniphila*	Therapeutic	Liver injury	Mice	Oral gavage	Not reported	Oral administration of *Akkermansia muciniphila* EVs for four weeks prevents liver injury in mice receiving high‐fat diet and CCL_4_ by enhancing intestinal integrity, inhibiting HSC activation and liver inflammation.	Raftar et al. (2022)
*Lactobacillus rhamnosus GG* (LGG)	Therapeutic	Alcoholic fatty liver disease (ALD)	Mice	Oral gavage	Not reported	LGG EVs protect the intestine from alcohol‐induced barrier dysfunction and alleviate liver steatosis and injury. LGG EVs also inhibit bacterial translocation and endotoxin release in ALD mice.	Gu et al. (2021)

An in‐depth characterization of FEVs is a prerequisite for meaningful safety and efficacy studies. FEVs are mostly reported below 500 nm in diameter with neutral to slightly negative surface charge (Tong et al., 2023; Wu et al., 2022; Zhang et al., 2016a). Different from EVs secreted from mammalian cells, the size of FEVs reported in the literature largely depends on the production technology. For instance, EVs obtained from various vegetables are very heterogenous in size, however, the isolation approaches such as those involving size‐exclusion chromatography column only yield EVs more homogeneous in size. Size may affect the stability of FEVs under digestive GI conditions and consequently their therapeutic efficacy (Tong et al., 2023). Whether the size and/or surface charge influence the bioavailability, biosafety and pharmacodynamics of FEVs remains unknown. Given that most isolation methods applied produce FEVs of a size ranging from 30 to 200 nm, our current knowledge of FEVs may be biased and therefore incomplete. This concern also applies to the composition of FEVs. Similar to cell‐derived EVs, FEVs contain large amounts of bioactive components, including proteins, nucleic acids and lipids, among which nucleic acids, in particular, miRNAs, are most extensively investigated. The contribution of proteins and lipids to the intrinsic bioactivities of FEVs remains largely unknown. In fact, even the miRNA contents of FEVs vary depending on the sources and the physiological state of their parent cells. Thus, it is probably challenging to identify active ingredients universally present in all types of FEVs. Instead, we may focus on the active ingredients and working mechanisms in the context of a specific disease. When employed as drug delivery systems, an in‐depth characterization of the physical and chemical properties as well as the composition of FEVs is similarly critical. Apart from potential impact on the bioavailability, biosafety and pharmacodynamics, the endogenous contents such as miRNAs may affect FEVs for their capacity to load and release exogenous compounds.

### 4.2 Administration routes and biodistribution

FEVs have been applied through various administration routes, and accordingly, FEVs present different biodistribution profiles.

#### 4.2.1 Oral administration

**FIGURE 4:**
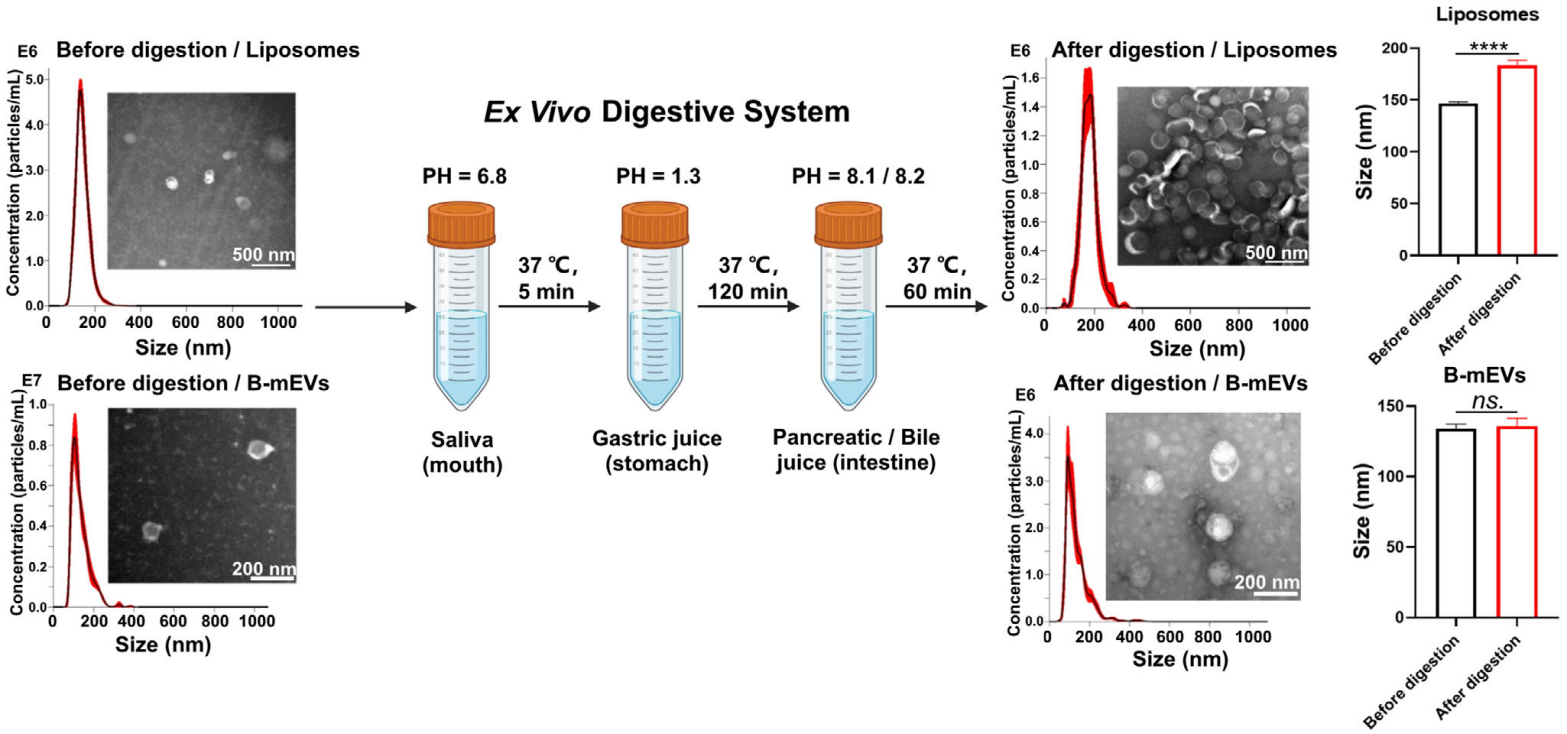
Milk‐derived extracellular vesicles (mEVs) survive the gastrointestinal environment. Morphology and size distribution of bovine mEVs (B‐mEVs) and liposomes before and after going through the ex vivo digestive system (Tong et al., 2023).

FEVs appear to be a suitable candidate for oral nanomedicine due to their resistance to the degrading GI conditions. For instance, milk‐derived extracellular vesicles (mEVs) can survive various harsh conditions in an ex vivo system that mimics different segments of the GI tract (Figure 4). After exposure to different conditions ranging from the mouth to the colon, mEVs remain intact while the integrity of other synthetic nanoparticles such as liposomes is damaged (Tong et al., 2023). Interestingly, smaller mEVs (<200 nm) are more resistant to the harsh GI conditions than larger ones (Tong et al., 2023). When boiled at 105°C for 15 min, mEVs remain intact whereas colorectal cancer cell‐derived EVs lose their integrity (Samuel et al., 2021). However, molecular mechanisms underpinning the resistance of mEVs to the harsh environment in the GI tract remain unclear. Compared to liposomes, EVs in general contain abundant sphingomyelin (SM) and cholesterol (Chol) (Subra et al., 2007) which bind each other via hydrogen bonds to form tight packing and detergent‐resistant SM/Chol bilayers (Kooijmans et al., 2012). In agreement, mEVs exhibit higher mechanical rigidity and more compact structure for the maintenance of stability under lower pH conditions (Wu et al., 2022). The highly rigid and stable SM/Chol bilayers may confer EVs with strong physical stability (de Gassart et al., 2003; Lu et al., 2018). Under acidic conditions and high temperature, calcium ions seem to enhance stability of mEVs (Samuel et al., 2021). Given that binding endogenous proteins such as albumin improves EV stability in the circulation (Liam‐Or et al., 2024; Liang et al., 2022; Liu et al., 2022), particularly the protein corona may facilitate the survival of FEVs when passing through the degrading GI conditions. Compared with EVs of other origins, it needs further investigation whether FEVs may have a unique structural design, such as a specific membrane composition of SM/Chol ratios or protein corona composition, constituting the superior stability of FEVs at the molecular level.

**FIGURE 5:**
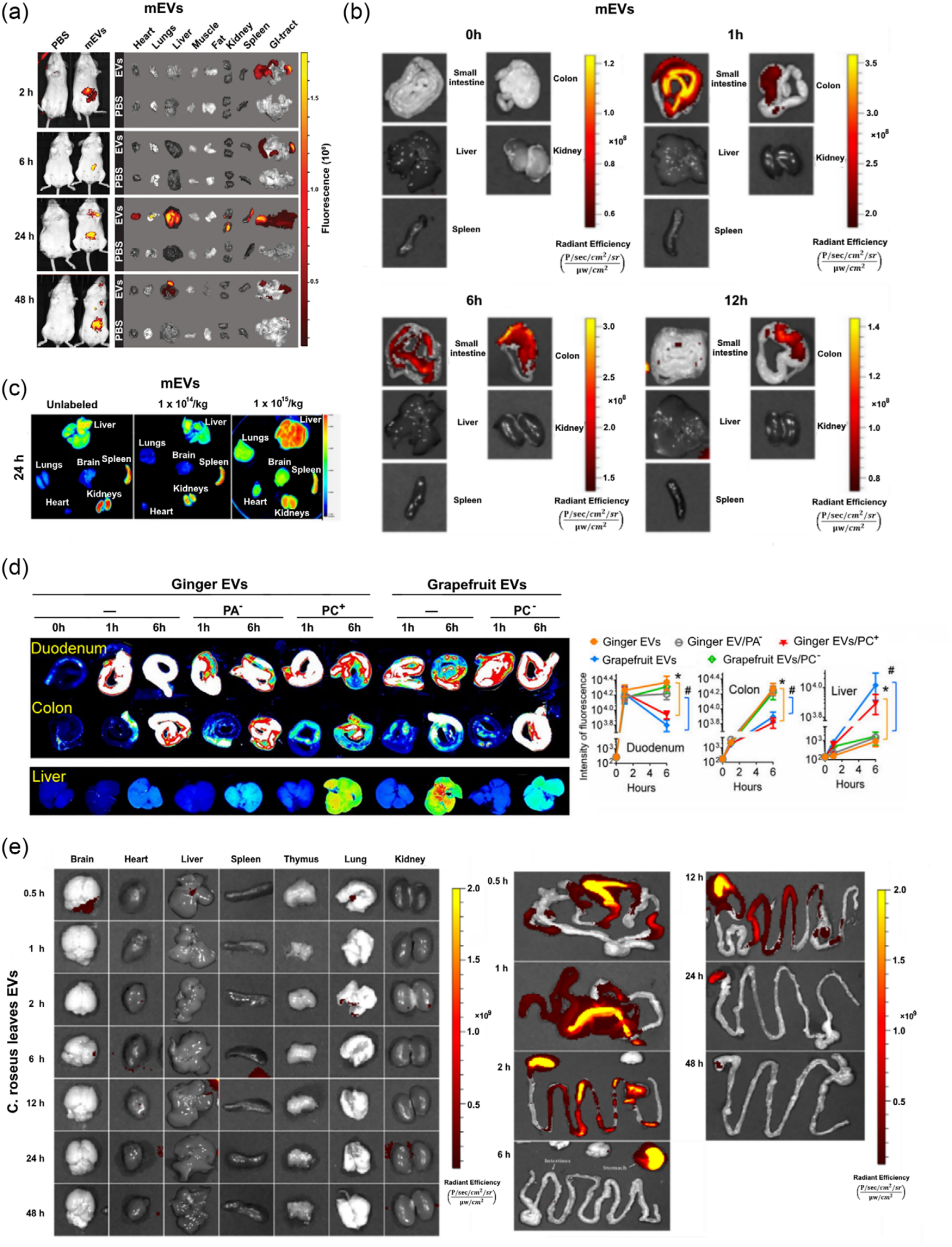
Biodistribution of FEVs following oral administration in mice. Bovine mEVs were administered at (a) 25 mg/kg (Samuel et al., 2021), (b) 15 mg/kg (Tong et al., 2021a), and (c) 10^14^ (≈130 mg/kg) or 10^15^ particles/kg (≈1300 mg/kg) (Manca et al., 2018). (d) Ginger EVs and grapefruit EVs were administered at 500 mg/kg (Teng et al., 2018). (e) *C. roseus* leaf EVs were administered at 60 mg/kg (Ou et al., 2023).

The biodistribution of FEVs following oral administration has been reported inconsistently in the literature. After oral administration in animals, mEVs have been reported to reach the colon and be retained there for a long time (Figure 5). Timewise, after oral administration, fluorescence dye DiR‐labelled mEVs arrive after 1 h in the small intestines and subsequently, at 6 h in the colon where mEVs stay for more than 12 h (Tong et al., 2021a). Beyond the gut, orally administered mEVs were reported to reach other organs, including the liver, spleen, kidney, pancreas, ovary, lung, heart and brain, as indicated by various labelling fluorophores (Betker et al., 2019; Manca et al., 2018; Samuel et al., 2021). However, our group did not detect fluorescence labelled mEVs in any other organs beyond the gut, neither in healthy nor diseased animals following oral gavage (Tong et al., 2020, 2021a, 2023). In agreement with our findings, a recent study in mice showed that mEVs were mainly in the GI tract and the fluorescence intensity of labelled mEVs in the liver was less than 0.3% of that in the gut (Khanam et al., 2023). This further supports our speculation that the proportion of mEVs reaching remote organs such as the liver is marginal, if any, following oral administration.

Similar to mEVs, plant‐derived EVs can survive the harsh GI environment to reach the colon and might be distributed to other organs when administered at a high dose (Figure 5d,e). For instance, orally‐administered grapefruit EVs and *Catharanthus roseus* leave EVs were mainly retained in the stomach and the gut (Ou et al., 2023; Wang et al., 2013). Similarly, broccoli‐derived EVs displayed an intestinal distribution profile upon oral administration but appeared to additionally target mesenteric lymph nodes and intestinal dendritic cells (Deng et al., 2017). Apart from the GI tract and mesenteric lymph nodes, EVs derived from ginger were observed in the liver upon oral gavage in mice (Zhuang et al., 2015). Moreover, EVs obtained from acerola juice were absorbed from the intestinal tract and observed in the liver and spleen (Umezu et al., 2021). The biodistribution of FEVs derived from probiotics seems similar to that of plant EVs. After 12 h of oral administration in mice, engineering probiotics‐derived membrane vesicles were mainly detected in the GI tract while weak signals were detected in the spleen and kidney (Liang et al., 2023).

The distribution profiles of FEVs shown by all those studies suggest that various types of FEVs can overcome the GI tract environment and reach the intestine. Upon arrival, different types of FEVs may be taken up by varied cell populations, such as macrophages, dendritic cells and epithelial cells, in the intestine (Tong et al., 2023; Umezu et al., 2021; Wang et al., 2014). However, whether oral administration of FEVs is able to reach remote organs such as liver and brain seems inconsistent in the literature. The discrepancies might be attributable to several reasons, including administration dose, labelling approaches, sample preparation protocols, and the time and methodology for detection. Of note, all the data available are based on the measurement of labelling chemicals but not FEVs per se. Yet, detailed studies are highly desirable as biodistribution is essential for understanding their mechanisms of action.

#### 4.2.2 Other administration routes

Biodistribution of FEVs was also reported following administration routes other than the oral route. In a study aimed at exploring the drug delivery potential of grapefruit EVs, the influence of administration routes on their biodistribution was comprehensively assessed (Wang et al., 2013). Seventy‐two hours after intravenous or intraperitoneal administration, the majority of DiR‐labelled grapefruit EVs were detected in the liver, lung, kidney and spleen. At the cellular level, after intravenous injection, grapefruit EVs were taken up in the spleen by DX5^+^ NK cells (10.9%) and F4/80^+^ cells (12.5%), and in the liver by F4/80^+^ cells (4.65%), DX5^+^ NK (1.75%) and CD19^+^ B cells (1.63%). Surprisingly, grapefruit EVs seem to have a long half‐life in vivo as DiR fluorescence was still detectable in the blood circulation after 7 days of intravenous administration and the fluorescent signal remained strong in the liver and spleen for up to 20 days. Following intramuscular injection, as expected, DiR‐labelled grapefruit EVs mainly stayed in the muscle. However, upon intranasal administration, grapefruit EVs accumulated primarily in the lung and brain, in particular in the brain, with label of grapefruit EVs staying up to 72 h after administration (Wang et al., 2013). In another recent study, radiative labelling was used to track mEVs in vivo with single photon emission computed tomography (SPECT) imaging (Gonzalez et al., 2020). In this study, mEVs were labelled with radioactive technetium (^99m^Tc). Similar to grapefruit EVs, mEVs administered through intravenous injection accumulated predominantly in the liver and spleen, whereas intraperitoneally injected mEVs tended to accumulate in the thyroid and stomach (Gonzalez et al., 2020). Compared to optical imaging after fluorescent dyes labelling exosomes (Wang et al., 2013), nuclear imaging techniques and SPECT may avoid the inherent background generated by natural biomolecules or the instability of the probe which leads to incorrect data resulting from the non‐specific signal of free dyes. Unfortunately, the oral administration route was not investigated by SPECT in Gonzalez's study. Although it is unclear whether those FEVs remain intact in the circulation or tissues even though the labelled fluorescence or radioactive signal is detectable, it is tempting to speculate that the long circulation of intact FEVs confers them with long‐lasting biological effects and suitability for drug delivery.

Based on the studies so far, FEVs are predominantly distributed to the GI tract following oral administration, to the liver and spleen following intravenous injection, and to several organs following intraperitoneal injection. Intramuscular and intranasal administration results in mainly local distribution. Of note, it seems that the biodistribution of FEVs can be detoured, to a marginal extent, by adjusting administration doses and timing. Regardless of administration routes, FEVs have demonstrated good safety. For instance, mEVs were well tolerated and caused little toxicity in vivo despite systemic distribution after injection. Mice did not develop allergic reactions after continuous injections of mEVs (Somiya et al., 2018). Likewise, no immune reactions, liver function impairment or erythropoiesis impairment was observed when mEVs were administered orally to rats, indicating their biosafety (Munagala et al., 2016; Ngu et al., 2023). However, mEVs might impose negative effects at extremely high dosages (Oliveira et al., 2016; Samuel et al., 2021). Nevertheless, from a practical perspective, oral administration is often considered advantageous over other routes of administration due to its convenient and non‐invasive nature. It allows for self‐administration and is particularly beneficial for patients who may encounter difficulty with other administration routes, such as injections. Oral administration can also provide a gradual release of the substance into the bloodstream, contributing to stable and prolonged therapeutic effects. For gut and gut‐related diseases, the oral administration route is particularly effective since it enables direct contact between the drug and the diseased sites (Applegate et al., 2022). Compared to other nanoparticles, FEVs are particularly suitable for oral administration due to the following reasons: (1) FEVs are derived from natural sources and have better safety and biocompatibility than synthesized vehicles; (2) Compared to cell‐derived EVs, FEVs resist the harsh conditions in the GI tract and (3) FEVs can crosstalk with gut microbiota, thus directly acting on the gut barrier or indirectly on the remote organs via bacterial metabolites. In light of these advantages of FEVs for oral administration and the presence of gut‐liver axis, FEVs hold great promise for their biomedical applications in ameliorating gut and liver diseases as well as other associated metabolic disorders via the gut‐liver axis.

### 4.3 Prebiotic effects in health

Orally administered FEVs can survive the gastric fluid and reach the intestine and therefore potentially impact the gut microbiota, gut barrier and the gut‐liver axis. FEVs carry many miRNAs, proteins and lipids from their parental cells, which empower FEVs to exert biological function in the host. In fact, the survival of orally administered plant‐derived miRNAs in the harsh GI environment is largely attributable to the protection of those miRNAs by plant EVs (Jia et al., 2021).

In light of their bioactive contents, FEVs may even benefit the healthy host. The regulating effect of mEVs on the intestinal flora has frequently been mentioned. Given the critical role of human milk in the establishment of the gut microbiota in infants, it was hypothesized and eventually discovered that human breast mEVs carry multiple bioactive cargoes which enable mEVs to regulate the gut microbiome and metabolites (Le Doare et al., 2018). Similar bioactivities have been reported for mEVs from many other animal species in recent years, which was summarized in a recent review (van Herwijnen et al., 2018). One interesting study on bovine mEVs showed that oral administration of mEVs altered bacterial communities in the murine cecum, including three phyla, seven families and 52 operational taxonomic units (Zhou et al., 2019). More specifically, we found that oral administration of mEVs promoted the growth of SCFAs‐producing microbes and increased the intestinal levels of SCFAs (Tong et al., 2020). In addition, oral administration of mEVs also increased plasma levels of IgA and enhanced host immunity (Tong et al., 2020). Similarly, plant‐derived EVs have been reported to be taken up by the gut microbiota and thereafter alter microbiome composition and host physiology with RNAs carried by those EVs (Teng et al., 2018). Further study indicates that miRNAs in ginger EVs regulate the communication between gut microbiota and the host immune system, resulting in a homeostatic balance between immunity and gut microbiota (Dad et al., 2021). miRNAs in EVs derived from ginger and grapefruit EVs may target the genes in gut probiotic *Lactobacillus rhamnosus* (LGG), thereby increasing the abundance of LGG and improving gut health (Dad et al., 2021).

### 4.4 Therapeutic potential in gut disorders

As expected, the most obvious therapeutic effects of orally administered FEVs are observed and extensively studied in various gut disorders, including IBD, malnutrition and colorectal cancer (Table 1). A common pathological characteristic of gut disorders is dysbiosis. As presented in Table 1, mEVs exert therapeutic effects on gut disorders such as IBD by regulating the abundance of gut microbiota and excretion of bacterial EVs. For example, oral administration of bovine mEVs regulates intestinal immune homeostasis and restores gut microbiota, thereby alleviating UC (Tong et al., 2021a). Moreover, oral administration of mEVs was reported to increase the abundance of *Dubosiella, Bifidobacterium, UCG*‐007*, Lachnoclostridium* and *Lachnospiraceae* genera in the colon (Zhou et al., 2019). Apart from mEVs, EVs derived from plants also potentially regulate gut microbiota and alleviate colon diseases. For probiotic EVs, several studies have shown that EVs derived from various types of probiotics, including LGG, *Lactobacillus plantarum* Q7 and *Clostridium butyricum*, have an effect on hindering colon shortening, colonic tissue damage and gut barrier disruption. Those therapeutic effects may be due to the restoration of gut microbiota by probiotic EVs (Hao et al., 2021; Ma et al., 2022; Tong et al., 2021b).

Another common feature of gut disorders is the leaky gut where the intestinal integrity is disrupted. Apart from regulating gut microbiota, FEVs also act on intestinal epithelial cells and immune cells, thus promoting gut barrier integrity. In return, the modulated gut barrier permeability can affect the cellular uptake efficiency of dietary metabolite, postbiotics, food‐derived miRNAs, FEVs and even bacteria‐derived EVs (Diez‐Sainz et al., 2021). It is noticeable that the gut barrier integrity protective effect of FEVs is usually related to anti‐inflammatory processes. For example, ginger EVs promote intestinal mucosa healing by reestablishing levels of pro‐ and anti‐inflammatory cytokines and the expression of multiple cytoplasmic/membrane proteins, thus alleviating acute UC and preventing chronic UC and UC‐associated cancer (Zhang et al., 2016a). Another study indicates that EVs extracted from citrus sinensis downregulate the expression of inflammatory cytokines while increasing the expression of tight junction proteins, thereby inhibiting inflammation and restoring the functionality of the intestinal barrier in IBD (Du et al., 2022). EVs extracted from other plants, such as grapes, kefir and lemon, seem to have similar therapeutic effects in IBD progression. Apart from plant EVs, mEVs also exert potent anti‐inflammation and protective effects on the gut barrier. In fact, mEVs have been reported to improve intestinal barrier integrity and intestinal architecture in mouse models of malnutrition and IBD (Maghraby et al., 2021; Tong et al., 2023).

### 4.5 Therapeutic potential in liver disease

Considering the impact of FEVs on gut microbiota and gut barrier integrity, two key factors affecting the gut‐liver axis, in gut disorders, the therapeutic effects of FEVs in liver disease may not be unexpected. In fact, the treatment efficacy of FEVs has been reported in various liver diseases, such as NASH, liver failure and ALD (Table 2).

FEVs predominantly accumulate in the liver following intravenous or intraperitoneal administration and therefore can exert direct effects on the liver. Given through i.v. injection, DiR‐labelled grapefruit EVs were predominantly detected in the liver at 72 h and the fluorescent signals remained strong at day 20 (Wang et al., 2013). In a mouse model of acute liver damage, intraperitoneally injected Shiitake mushroom EVs could alleviate liver injury, as evidenced by downregulated IL‐6, ALT and AST levels, via inhibiting NLRP3 inflammasome activity (Liu et al., 2020a). Similarly, in the same disease model, EVs derived from honey exert liver protective effects following intraperitoneal administration in mice through inhibition of the formation and activation of the nucleotide‐binding domain and leucine‐rich repeat‐related (NLR) family (Chen et al., 2021). All those studies demonstrate that FEVs can inhibit hepatic inflammation directly to protect the liver from acute injury.

**FIGURE 6:**
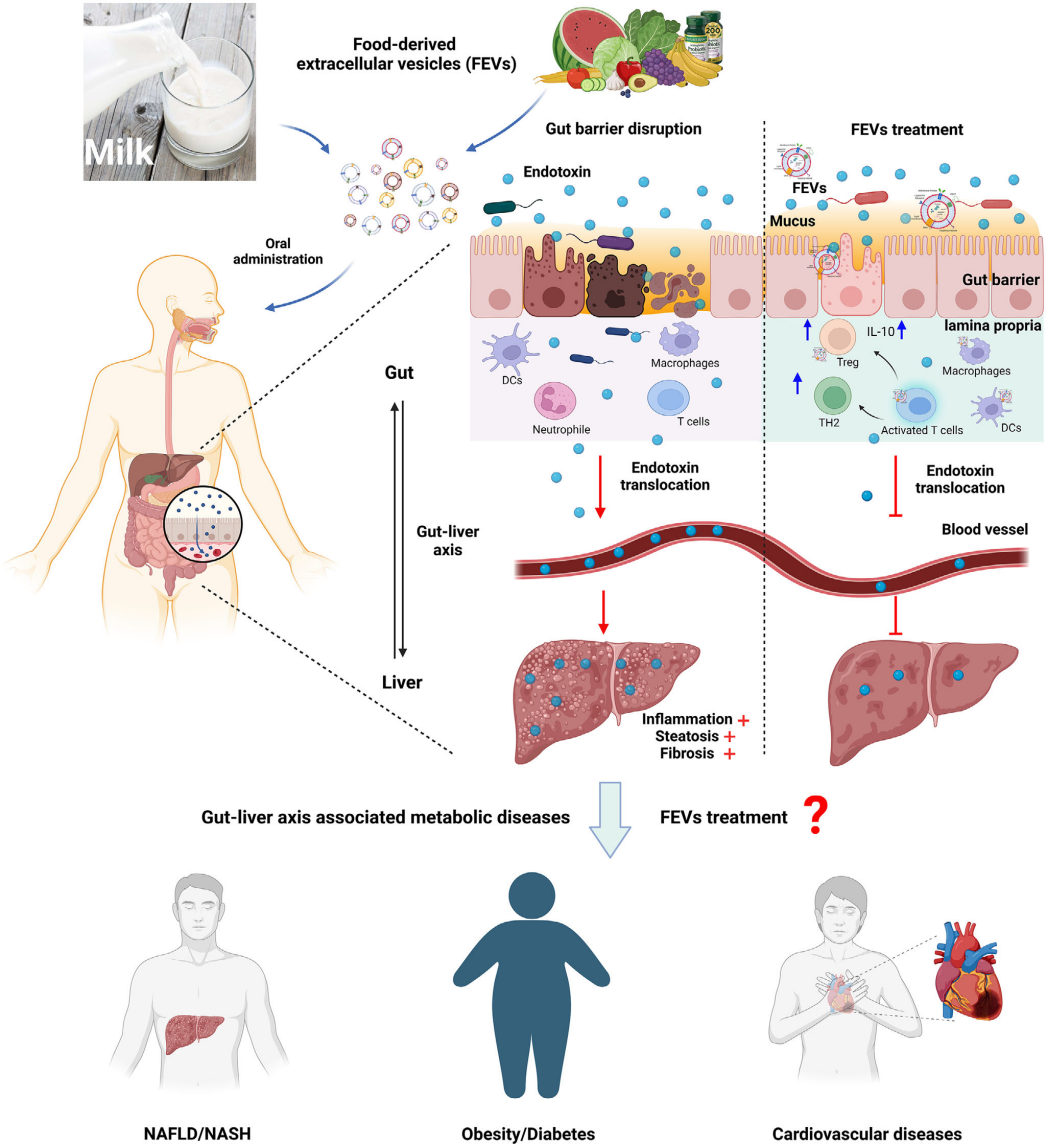
FEVs modulate metabolic diseases via the gut‐liver axis. A leaky gut permits translocation of gut bacterial endotoxins into the bloodstream and subsequently remote organs such as the liver, thereby inducing or deteriorating disease progression therein via the gut‐liver axis. In turn, via the gut‐liver axis, diseases related to the remote organs may cause dysbiosis and worsen the leaky gut. We now propose that FEVs can act locally in the gut by modulating intestinal barrier integrity and microbiota to remotely impact the liver and elicit therapeutic effects via the gut‐liver axis. Since the liver is the metabolic hub, we presume that FEVs also exert therapeutic effects in other metabolic diseases such as obesity, diabetes and cardiovascular disease.

In contrast to acute liver injury, oral administration of FEVs is preferable for the treatment of chronic liver conditions. As discussed earlier, if FEVs primarily stay in the GI tract following oral administration, it may be hard to achieve a therapeutic dose in the liver. In fact, our recent study demonstrates that oral administration of mEVs is able to effectively migrate liver steatosis and inflammation in NASH despite lack of detectable mEVs in the liver (Tong et al., 2023). Given that FEVs can influence the growth and activity of gut microbiota and regulate gut barrier integrity, orally delivered FEVs likely impact the liver through the gut remotely, that is, the gut‐liver axis (Figure 6). This concept is supported by several recent studies where oral administration of FEVs results in liver protective effects in various chronic liver conditions such as fatty liver disease.

For chronic liver injury, two studies showed that oral gavage of EVs derived from *Akkermansia muciniphila* could reduce serum inflammatory cytokines and alleviate liver and colon damage in a mouse model of high fat diet and CCl_4_‐induced liver injury (Raftar et al., 2021, 2022). In a mouse model of alcohol‐induced liver damage, oral administration of ginger EVs activates nuclear factor erythroid 2‐related factor 2 (Nrf2) and thereby enhancing liver detoxification and antioxidant capability (Zhuang et al., 2015). Notably, Shogaols, dehydrated analogues of the gingerols, carried in the ginger EVs account for the activation of Nrf2 through a pathway dependent on TLR4/TRIF signaling (Zhuang et al., 2015). Although a significant amount of ginger EVs were detected in the liver and the liver protection may be largely attributable to the direct effects of those vesicles on the hepatocytes, indirect effects via the gut‐liver axis cannot be excluded as ginger EVs also benefit the intestinal wall in the gut (Mu et al., 2014).

Fatty liver disease is a general term for chronic liver conditions, including ALD and NAFLD, both of which are associated with gut health status via the gut‐liver axis (Arab et al., 2020). Hence, apart from directly acting on the liver, FEVs may exert their liver protective effects via acting on the gut microbiome and gut barrier in fatty liver disease. For example, upon oral administration, EVs originating from the probiotics, LGG exhibited a safeguarding effect on the intestinal barrier against alcohol‐induced gut barrier dysfunction thereby curtailing translocation of gut bacteria and endotoxins to the liver, which shielded the liver from both steatosis and injury in an animal model of ALD (Gu et al., 2021). Apart from ALD, FEVs have been reported to benefit NASH, a severe form of NAFLD. Our recent study demonstrates that oral administration of mEVs reinstates the integrity of the gut barrier and prohibits translocation of endotoxins into the liver, as a result, effectively mitigating liver inflammation and progression of NASH (Tong et al., 2023).

Apart from fatty liver disease, the benefit of FEVs given by oral gavage on liver fibrosis and cirrhosis was also reported. For instance, EVs derived from tea leaves suppress the activation of hepatic stellate cells in a CCL_4_‐induced liver fibrosis model (Gong et al., 2022). In a mouse model of liver fibrosis/cirrhosis, oral administration of mEVs curtailed proliferation and activation of hepatic stellate cells, as evidenced by the reduced expression of alpha‐smooth muscle actin and collagen type I, and therefore attenuated liver fibrosis (Reif et al., 2022).

### 4.6 Therapeutic potential in other metabolic diseases

Since the liver is a hub metabolically connecting to various tissues, FEVs may therefore impact other metabolic diseases, such as obesity, T2D and CVD (Figure 6). The evidence regarding the role of FEVs in these metabolic diseases is limited and not as much studied as in the field of liver disease. Nonetheless, some preliminary findings are promising. In general, FEVs exert therapeutic effects on non‐hepatic metabolic diseases through anti‐inflammatory and anti‐oxidative pathways. In mouse models of obesity, garlic EVs given through oral gavage inhibited body weight increase, reduced glucose tolerance and enhanced insulin sensitivity (Liu et al., 2021a). Interestingly, obesity‐induced brain inflammation was also attenuated by oral administration of garlic EVs via the gut‐brain axis (Sundaram et al., 2022). In another mouse model of obesity, orally administered orange EVs accumulated in the intestinal region that is involved in dietary lipid absorption (Berger et al., 2020). After 1 month of treatment, villi size was increased and expression of lipid absorption‐related genes was modulated, thus alleviating obesity (Berger et al., 2020). Human breast milk EVs can regulate infant body weight and body composition. Human mEVs RNA components, especially miR‐148a, were shown to negatively regulate the body weight and fat mass of infants (Shah et al., 2021). Apart from obesity, some studies also revealed the regulatory effects of FEVs on T2D. EVs from *Akkermansia muciniphila*, a next‐generation probiotic, were tested in high fat diet‐induced diabetic mice. Oral gavage of those probiotic EVs for 5 weeks, tissue inflammation and insulin resistance were reduced in the mice, likely due to enhancement of intestinal tight junction function (Ashrafian et al., 2019). The therapeutic potential of FEVs on CVD was evidenced by a recent study where carrot EVs markedly hindered ROS generation and cell apoptosis in cardiomyoblast cells (Kim and Rhee, 2021).

Although studies are limited, the therapeutic potential of FEVs has been demonstrated in both GI disorders and various metabolic diseases. Mechanistically, FEVs may function either directly on the site of disease or indirectly via the gut‐liver or gut‐brain axis or other pathways. Nonetheless, those early but promising preclinical findings have already formed concepts for clinical translation in the future.

### 4.7 Therapeutic potential as oral drug delivery systems

Apart from being potential therapeutics per se attributed to their intrinsic activities, FEVs may serve as natural drug delivery vehicles, in particular, for oral administration. So far, several synthetic nanoparticle systems have been explored for oral drug delivery in ulcerative colitis (UC) (Chen et al., 2023). For instance, Zhuo et al. developed a hyaluronic acid (HA)‐modified poly (lactic‐co‐glycolic acid) (PLGA) nanoparticle system to deliver bilirubin (Zhuo et al., 2024). This HA‐PLGA system had an encapsulation efficiency of 47.6% ± 2.7% and released more bilirubin in simulated intestinal fluid (pH 7.4) than that in simulated gastric fluid (pH 1.2). The pH‐sensitive PLGA system has also been explored for delivery of curcumin (Beloqui et al., 2014) and small RNAs (Frede et al., 2016; Wang et al., 2023a). The pH‐sensitive liposome system is another nanoparticle system explored for oral drug delivery. Aib et al. developed a co‐polymer eudragit‐S100 coated pH‐sensitive liposome system for co‐delivery of mesalazine and curcumin in a guinea pig model of UC (Aib et al., 2022). This liposome system shows an encapsulation efficiency of 78.3% ± 4.2% and achieves a high drug release profile (88%) at intestinal pH 7.4 with a very low leakage of payload (7%) at pH 1.2. For most synthetic oral drug delivery systems, coating polymers are employed to prevent drug leakage in the gastric fluid. In contrast, as natural oral drug delivery systems, FEVs per se can withstand the degrading GI conditions. Similar to synthetic oral drug delivery systems, FEVs are mostly used to deliver drugs for the treatment of gut diseases such as UC. Ginger EVs were reported to achieve an extremely high encapsulation efficiency of doxorubicin (Dox), up to 95.9% and to release loaded Dox in a pH‐dependent manner in comparison with commercially available liposomal Dox (Zhang et al., 2016b). Among all the FEVs, mEVs are the most widely studied system for oral delivery of therapeutics (Adriano et al., 2021). With a sonication approach, insulin was encapsulated into mEVs with an efficiency of 15.9% and achieved successful reduction of glucose levels following oral administration (Wu et al., 2022). Although the encapsulation efficiency is lower than in the case of synthetic nanoparticles, mEVs enable a sustained release of insulin. mEVs have also been explored for oral delivery of siRNAs. Electroporation is often used but yields varied encapsulation efficiency and the release profile is not reported (Aqil et al., 2019; Han et al., 2024). So far, FEVs have shown promise in oral drug delivery, however, compared with synthetic nanoparticle delivery systems, the drug loading efficiency and release dynamic profiles of FEVs have not been well characterized. Moreover, the targeting efficiency of FEVs, either via passive or active targeting, is poorly studied. Similar to conventional drug delivery systems, the drug delivery capabilities of FEVs may be improved by biochemical engineering approaches such as surface modifications.

Due to their natural origin, FEVs are considered to impose lower safety risk and less toxicity compared to other drug delivery systems, especially for oral administration. Since FEVs survive the degrading conditions in the GI tract, they can protect their cargo, either naturally inherited from their food origin or artificially encapsulated, from degradation and thereby delivering the cargo to various segments of the intestine. Likewise, one would expect that FEVs can deliver drugs and biological compounds for the treatment of GI diseases or metabolic disorders via the gut‐liver axis. Nonetheless, either as therapeutic agents or delivery carriers, potential applications of FEVs have been explored in various disease conditions (Tables 1 and 2). In light of promising preclinical data, FEVs have sparked tremendous clinical interest in recent years.

## 5 CLINICAL TRANSLATION

To date, research on FEVs is still at an infant stage. There are no FDA‐approved FEVs for therapeutic purposes so far but several FEVs have been registered in clinical trials (Table 3). Since FEVs are replete with bioactive small molecules, proteins, RNAs, and other physiologically and pharmacologically active compounds, their intrinsic therapeutic potential has been tested in human subjects. For instance, FEVs derived from grapes were registered to avert oral mucositis that is associated with chemoradiation therapy for head and neck cancer (ClinicalTrials.gov: NCT01668849) (Wu et al., 2017). Moreover, FEVs derived from aloe and ginger are in human clinical trials to treat and mitigate symptoms associated with polycystic ovary syndrome (ClinicalTrials.gov: NCT03493984). Apart from acting as therapeutic agents per se, FEVs have also been registered in clinical trials as drug delivery vehicles of curcumin for the treatment of colon tumours (ClinicalTrials.gov: NCT01294072) (Wu et al., 2017). Although clinical trials on FEVs remain scarce and the conclusions of known clinical trials are not yet available (https://clinicaltrials.gov/), FEVs are emerging as a candidate for next‐generation oral nanomedicine, in particular, for the treatment of gut diseases and gut‐liver axis‐associated metabolic disorders.

**TABLE 3:** Registered clinical trials of FEVs.

FEV source	Function	Indication	Clinical trial number	Start date	End date	Status
Fruit	Drug delivery vehicles	Colon cancer	NCT01294072	2011‐01	2024‐11	Ongoing
Grapes	Therapeutic	Oral mucositis	NCT01668849	2012‐08	2022‐06	Completed
Ginger	Therapeutic	IBD	NCT04879810	2018‐03	2022‐08	Completed
Aloe vera and ginger	Therapeutic	Polycystic ovary syndrome	NCT03493984	2019‐10	2020‐05	Withdrawn
Citrus limon	Therapeutic	Metabolic syndrome	NCT04698447	2021‐02	2019‐03	Ongoing

## 6 CHALLENGES AND PERSPECTIVES

FEVs, derived from various sources of food, resemble other types of nanoparticles in terms of morphology and size and even their in vivo behaviour to a certain extent. Moreover, FEVs, as a product of natural origin, offer multiple advantages such as low cytotoxicity, cost‐effectiveness, source accessibility and high tolerance to the harsh degrading GI environment, making them a promising candidate for oral nanomedicine. The advantage of using FEVs over their respective food sources, besides enrichment of active ingredients, is the possibility to remove potential adverse components, such as lactose in the milk. To date, promising therapeutic effects of FEVs have been observed in disease models, however, the mechanisms of action are still a matter of speculation. Given that the majority of orally administered FEVs remain in the gut and interact with the intestinal barrier and gut microbiota, we propose that FEVs, either as therapeutic agents or drug delivery systems, impact remote organs via the gut‐liver axis following oral administration. Accordingly, FEVs may be feasible for long‐term use in chronic conditions such as IBD and NASH as well as other metabolic disorders.

Despite the vast promise, clinical translation of FEVs faces various challenges: (1) It is difficult to standardize and industrialize the source of EVs. Since FEVs are extracted from plants, milk, honey or probiotics, the source of FEVs varies from batch to batch. Moreover, approaches for characterization of FEVs are not standardized. Currently, the main methods are still dynamic light scattering (DLS) and nanoparticle tracking analysis (NTA) which both rely on Brownian Motion to calculate EV size. However, both techniques suffer from a drastic reduction in the ability to visualize small particles in polydisperse samples and a limited ability to discriminate EVs of different sizes (Bachurski et al., 2019; Erdbrugger & Lannigan, 2016). (2) A common challenge in FEV research is the poor definition of active pharmaceutical ingredients (API). Varied sources and quality control complicate this issue even further. miRNAs are the most widely reported APIs in FEVs likely due to the technical readiness to sequence miRNAs and to map their downstream pathways with available databases as well as to synthesize specific miRNAs for functional validation. (3) Up to now, the mechanisms of action following different administration routes and in various contexts of diseases remain unclear. This is largely due to the complexity of the bioactive contents in FEVs and the challenge of singling out individual components for any specific therapeutic activities, which is further complicated by the multiple biological pathways impacted by FEVs. These difficulties may explain why the APIs of FEVs were not reported in most studies (Tables 1 and 2). This is particularly true for FEVs given by oral administration. As a result, (4) it is challenging to determine the therapeutic dosage of FEVs. Thus, similar to mesenchymal stromal cell‐derived small EVs (Gimona et al., 2021), potency tests for therapeutic applications of FEVs should be developed. (5) There is a lack of sophisticated protocols for reproducible large‐scale production of FEVs, thus hindering their potential clinical applications. Currently, the primary extraction approach for FEVs is pretreatment of food materials followed by ultracentrifugation, which is notoriously tedious and requires special equipment. Given the huge efforts have been made for manufacturing therapeutic human cell‐derived EVs in large‐scale (Gimona et al., 2021, 2017; Ma et al., 2024), such as polymer precipitation (Borger et al., 2020; Wang et al., 2017) and tangential flow filtration approaches, similar strategies may be referred for manufacturing therapeutic FEVs. (6) To develop FEVs for drug delivery, apart from the challenges that have been summarized for non‐food EVs (Escude Martinez de Castilla et al., 2021), the heterogeneous size of FEVs may affect their drug loading efficacy and pharmacokinetic profiles in vivo (Lian et al., 2022). (7) There is limited information on storage conditions which are critical for commercialization and clinical applications. (8) Clinical translation of FEVs may face concerns of regulatory affairs as there are no precedent pharmaceutical approvals yet.

To address the challenges and facilitate the clinical translation of FEVs, we propose some practical solutions as follows. To avoid variations of FEVs and increase experimental reproducibility, more detailed information on the food source origins, such as the colostrum or mature stages of milk for mEVs, should be well documented and reported in all studies. More elegantly designed experiments for in‐depth studies are needed to identify active ingredients and understand the mechanisms of action of various FEVs in specific disease contexts. To develop FEVs into therapeutics or drug delivery systems, more systemic efforts, including better biodistribution studies with firm labelling methods, are highly needed. Given that the observed therapeutic responses may be confounded by some non‐EV proteins, RNAs or other components from the food sources of FEVs, we propose to use EV‐free supernatant from the respective food sources, instead of saline, as the control (or placebo) for FEVs in (pre)clinical studies (Tong et al., 2023). Immediately next to this is the purity of FEVs. Additional processes to eliminate bioactive compounds that are not carried but loosely associated with FEVs may be applied while preserving the size, morphology and biological functions of FEVs. Emerging new extraction strategies such as size‐exclusion chromatography column‐mediated isolation may be useful. Cost‐effective large‐scale production approaches and a robust quality control framework are imperative to minimize batch‐to‐batch variations in FEVs. To address this demand, the massive effort and progress on scalable and reproducible manufacturing processes of EVs derived from mesenchymal stem cells (Borger et al., 2020; Gimona et al., 2017; Pachler et al., 2017) are worth referring to. Shelf storage conditions should be optimized to lay the foundation for their clinical applications. Moreover, certain bioactive compounds in FEVs might pose perils even though they are derived from food sources, thus, potential toxicity shall be cautiously assessed.

Yet, many outstanding research questions on FEVs remain, such as:

- Are FEVs more stable than other EVs derived from mammalian cells in the degrading GI environments?
- In terms of resilience, what constitutes the unique superiority of FEVs over other EVs and synthetic nanoparticles at the molecular level? Does EV membrane structural composition, surface charge, size or protein corona contribute to the superior stability of FEVs?
- What determines the biodistribution of FEVs following oral administration? Why inconsistent observations of biodistribution are reported even on the same FEVs?
- What are the active ingredients in FEVs and is there a common mechanism of action of FEVs for their therapeutic effects? More in‐depth multi‐omics analysis may help.
- Can FEVs impose any adverse effects, and if so, under what conditions?
- How to improve drug loading efficiency into FEVs?
- What are the unique biochemical properties and specific cargo that determine the safety and drug delivery efficiency of FEVs? More in‐depth knowledge of FEVs’ composition will support the downstream choice of the best‐suited delivery system in a disease‐specific manner.
- How do FEVs function, either as therapeutic agents or drug delivery systems, via the gut‐liver axis?

Nevertheless, FEVs are natural and edible nanoparticles of great potential for future nanomedicine. Although their molecular mechanisms of action need to be further defined, emerging evidence suggests that FEVs can be used to develop more efficacious treatment strategies for GI disorders and various metabolic diseases that involve the gut‐liver axis. Therefore, much more efforts to develop FEVs‐based therapeutics drug delivery systems are anticipated in the coming years.

## AUTHOR CONTRIBUTIONS

Jiong‐Wei Wang: Conceptualization; data curation; formal analysis; funding acquisition; supervision; validation; visualization; writing—original draft; writing—review and editing. Sitong Zhang: Data curation; formal analysis; visualization; writing—original draft; writing—review and editing. Qiyue Wang: Formal analysis; visualization; writing—original draft; writing—review and editing. Cheng Han Ng: Formal analysis; visualization; writing—original draft; writing—review and editing. Yan Xian: Formal analysis; visualization; writing—original draft; writing—review and editing. Dan Li: Formal analysis; funding acquisition; writing—original draft; writing—review and editing. Gert Storm: Formal analysis; funding acquisition; writing—original draft; writing—review and editing. Lingjun Tong: Data curation; formal analysis; visualization; funding acquisition; supervision; writing—original draft; writing—review and editing.

## CONFLICT OF INTEREST STATEMENT

The authors declare that they have no known competing financial interests or personal relationships that could have appeared to influence the work reported in this paper.
